# Regularities in vertical saccadic metrics: new insights, and future perspectives

**DOI:** 10.3389/fpsyg.2023.1157686

**Published:** 2023-05-12

**Authors:** Harold H. Greene, Vaibhav A. Diwadkar, James M. Brown

**Affiliations:** ^1^Department of Psychology, University of Detroit Mercy, Detroit, MI, United States; ^2^Department of Psychiatry and Behavioral Neurosciences, Brain Imaging Research Division, Wayne State University, Detroit, MI, United States; ^3^Department of Psychology, University of Georgia, Athens, GA, United States

**Keywords:** saccade programming, vertical saccades, inhibition, fixation duration, asymmetry

## Abstract

**Introduction:**

Asymmetries in processing by the healthy brain demonstrate regularities that facilitate the modeling of brain operations. The goal of the present study was to determine asymmetries in saccadic metrics during visual exploration, devoid of confounding clutter in the visual field.

**Methods:**

Twenty healthy adults searched for a small, low-contrast gaze-contingent target on a blank computer screen. The target was visible, only if eye fixation was within a 5 deg. by 5 deg. area of the target’s location.

**Results:**

Replicating previously-reported asymmetries, repeated measures contrast analyses indicated that up-directed saccades were executed earlier, were smaller in amplitude, and had greater probability than down-directed saccades. Given that saccade velocities are confounded by saccade amplitudes, it was also useful to investigate saccade kinematics of visual exploration, as a function of vertical saccade direction. Saccade kinematics were modeled for each participant, as a square root relationship between average saccade velocity (i.e., average velocity between launching and landing of a saccade) and corresponding saccade amplitude (Velocity = S*[Saccade Amplitude]^0.5^). A comparison of the vertical scaling parameter (S) for up- and down-directed saccades showed that up-directed saccades tended to be slower than down-directed ones.

**Discussion:**

To motivate future research, an ecological theory of asymmetric pre-saccadic inhibition was presented to explain the collection of vertical saccadic regularities. For example, given that the theory proposes strong inhibition for the releasing of reflexive down-directed prosaccades (cued by an attracting peripheral target below eye fixation), and weak inhibition for the releasing of up-directed prosaccades (cued by an attracting peripheral target above eye fixation), a prediction for future studies is longer reaction times for vertical *anti-saccade* cues above eye fixation. Finally, the present study with healthy individuals demonstrates a rationale for further study of vertical saccades in psychiatric disorders, as bio-markers for brain pathology.

## Introduction

Visual information is spatially distributed, and has to be sampled in sequence. For seeing animals, shifts in gaze (i.e., where looking is directed) are necessary for optimal functioning in the environment. In vertebrates, gaze shifts are accomplished by head and eye movements. The combination of these movements for gaze shifts depends on structural constraints, and ecological function (see Land, 2015, 2019 for reviews). For example, birds, because of their light heads and flexible necks, prioritize moving their heads in a ballistic (i.e., saccadic) manner over making saccades (i.e., ballistic eye movements). When a bird’s head is restrained, saccades tend to have greater than usual amplitudes. Compared to birds, the primate head is heavier and the neck less flexible. Healthy humans for example, prioritize saccades overhead movements for small (25^o^) gaze shifts, with incremental contribution from the head (sometimes needlessly) for increasing amplitude shifts (Freedman, 2008; Franchak et al., 2021). In effect, saccades dominate gaze shifts within 25^o^ of eye fixation (i.e., the foveal, parafoveal and near peripheral visual field).

Functional differences exist for saccade metrics between healthy volunteers and volunteers with various psychiatric disorders (see Bittencourt et al., 2013 for a review). Hence, it is important to determine how saccades function in healthy brains, in order that variations observed in disordered brains may be utilized as biomarkers for disorder. Much of what is known about saccade mechanisms comes from constrained saccadic tasks, wherein the observer is instructed to execute a saccade to (i.e., pro-saccade), or away from (i.e., anti-saccade) a visual saccade cue (e.g., Pratt and Trottier, 2005), or is instructed to execute a saccade to the remembered location of a previously presented saccade cue (e.g., Abegg et al., 2015). The high level of experimental control in these constrained saccadic tasks makes them attractive for describing and modeling saccade mechanisms. However, mechanisms of self-paced, self-directed visual exploration may not be fully indexed by these constrained tasks. During visual exploration, saccades are typically self-paced and self-directed, as in the case of searching for a target object in the visual field. Polar distributions are useful for describing directional attributes of saccade metrics, and a productive approach for characterizing polar distributions is to quantify asymmetries in their shapes (Zhou and King, 2002; Foulsham and Kingstone, 2010; Greene et al., 2014). Generally, asymmetries in processing by the healthy brain demonstrate regularities that facilitate the modeling of brain operations. In the present context, asymmetries in the polar distribution of saccade metrics may provide increased diagnostic specificity for treatment of brain disorders characterized by impaired saccadic control. The present work focused on saccadic asymmetries along the *vertical meridian*, as they may be rooted in differential regularities of information experienced near the upright primate torso (i.e., below eye fixation) and in far space above eye fixation (e.g., Previc, 1990; Zhou and King, 2002; Greene et al., 2014).

Metrics associated with the direction of saccades during visual exploration, are unfortunately confounded by the configuration of clutter in the visual field. For example, while the probability of up-directed saccades is higher than down-directed ones when there is a presumption that a scene is upright (Foulsham et al., 2008; Foulsham and Kingstone, 2010; Greene et al., 2010, 2014), the direction of asymmetry changes with scene rotation (Foulsham et al., 2008; Foulsham and Kingstone, 2010). For saccade amplitudes, Greene et al. (2010) found longer down-directed than up-directed saccades when observers searched for a target symbol on road maps. However, no asymmetry was apparent in Greene et al.’s (2012) study, where observers searched for a target in a 25-item display, set on a 5 × 5 hexagonal matrix. In effect, scene properties guide visual exploration, and influence the polar distribution of *where* the eyes are directed (i.e., probability and amplitude of a saccade). Another metric of interest is how fast the eye moves to where it is directed. Results from constrained saccadic tasks suggest that this question is worth investigating. For example, Zhou and King (2002) found faster up-directed saccades for 2 macaque monkeys. However, human findings indicate slower (not faster) up-directed saccades than down-directed saccades (e.g., Bonnet et al., 2013; Tiadi et al., 2014). To date, we are not aware of any study that has determined the velocity of vertical saccades during self-paced, self-directed visual exploration of a visual field. Finally, for a comprehensive modeling of saccadic operations, it is necessary to determine *when* saccades are *initiated*. A systematic up-down asymmetry is typically reported for saccade latency in constrained saccadic tasks (Abegg et al., 2015; see also Greene et al., 2014 for a summary). Interestingly, the asymmetry has not been reported in lifespan studies that utilized participants between preschool and octogenarian ages (Bonnet et al., 2013; Irving and Lillakas, 2019). Although these studies utilized large sample sizes (i.e., more than 70 participants), statistical power was likely low from an insufficient numbers of stimulus trials. For example, each participant in Bonnet et al.’s (2013) prosaccade experiment executed only 8 saccades upwards, and downwards. Irving and Lillakas (2019) reported using different numbers of blocks of trials for participants, but did not report the number and direction distribution of trials in each block. In experimental studies of psychology, the number of repetitions (i.e., trials) per participant is an important component of statistical power (e.g., Baker et al., 2021). Despite few reports to the contrary, the evidence for shorter latency for up-directed saccades in healthy adults, is convincing. The asymmetry persists for pre-saccadic fixation duration for visual exploration, irrespective of scene property (see Greene et al., 2020 for a meta-analysis).

In summary, the present issue of concern is up-down saccadic asymmetries for self-paced, self-directed visual exploration. Scene configuration (e.g., rotation) influences up-down asymmetry for how often, and how far the eyes are directed (e.g., Foulsham et al., 2008; Foulsham and Kingstone, 2010). To date, vertical asymmetry in how fast the eyes move up or down, is an open question. With respect to saccade timing, the time to initiate a saccade is typically shorter for up-directed saccades. One goal of the present study was to determine asymmetries in saccadic metrics during visual exploration, devoid of confounding clutter in the visual field. This was accomplished by utilizing gaze-contingent eye-tracking technology. Specifically, asymmetries in pre-saccadic fixation duration, saccade amplitude, probability of directing a saccade, and saccade average velocity (i.e., average velocity between launching and landing of a saccade) were determined as a function of saccade direction. Saccade kinematics (specifically, average saccade velocity as a function of saccade amplitude) were also determined. A second goal was to introduce a theory of vertical saccade inhibition, towards providing new insights, and future perspectives on the study of saccades.

## Methods

### Participants

No study has conducted visual exploration of a clutter-free visual field, in the manner of the present study. Previous work (Greene et al., 2014) suggests that effect sizes for multi-direction analyses of saccade metrics produce ηp^2^ effect sizes of at least 0.10. Greene et al. (2014) utilized 12 participants (408 trials) to 44 participants (10 trials) in various experiments. In the present study, to detect an effect size, ηp^2^ of about 0.10 with 95% power, alpha = 0.05, and non-sphericity correction = 1, in an 18-level one-way repeated measures ANOVA (see results section), G*Power software calculations indicated the use of a sample size of about 18 participants.

Twenty adults (12 female) with no known neurological impairment provided informed consent to participate in the study. All procedures were approved by the Institutional Review Board at the University of Detroit Mercy, and the study was conducted in accordance with the guidelines in the Belmont Report. Participants were between 18 and 40 years of age (mean age was 22 years ±6). All had normal or corrected-to-normal visual acuity (i.e., at least 20/40). Nineteen participants had normal contrast sensitivity (CS) levels of at least 1.65, and one had an acceptable level of 1.50 Log CS units. All participants were unaware of gaze-contingent techniques in the viewing of displays. They were naïve about the study, and were paid $8.00 USD for their time.

### Stimuli and apparatus

The stimulus was a low contrast target (i.e., two 2% contrast dots separated by 0.8°) on a gray 50 cd/m^2^ background, as measured by a Konica Minolta LS − 110 luminance meter on a 17-inch monitor (1,024 × 768 pixels,75 Hz refresh rate). From the participant’s perspective, the monitor constituted a 25° vertical, 35° horizontal, and 40° diagonal search space. Contrast sensitivity of participants was measured using the Hamilton-Veale test. During visual exploration, eye position was sampled at 500 Hz by an Eyelink II eye tracker controlled by EYETRACK gaze-contingent functions.^1^ A saccade was recorded when eye velocity exceeded 30° s^−1^, or when eye acceleration exceeded 8,000° s^−2^. Periods of eye stability between saccades (as defined) were counted as fixations. The gaze-contingent code drew a background bitmap (i.e., gray screen) and a foreground window bitmap (i.e., target, in a randomly chosen location around imaginary concentric circles on a white screen). As the eye was tracked, the code instantaneously (about 14 ms delay) redrew those parts of the display that changed because of the 5^°^ × 5^°^ foveal window’s gaze-contingent movement. From the perspective of participants, the screen was blank unless the invisible moving foveal window overlapped the location of the target. Participants acknowledged finding the target by pressing the computer’s left mouse key.

### Procedure

**Figure 1:**
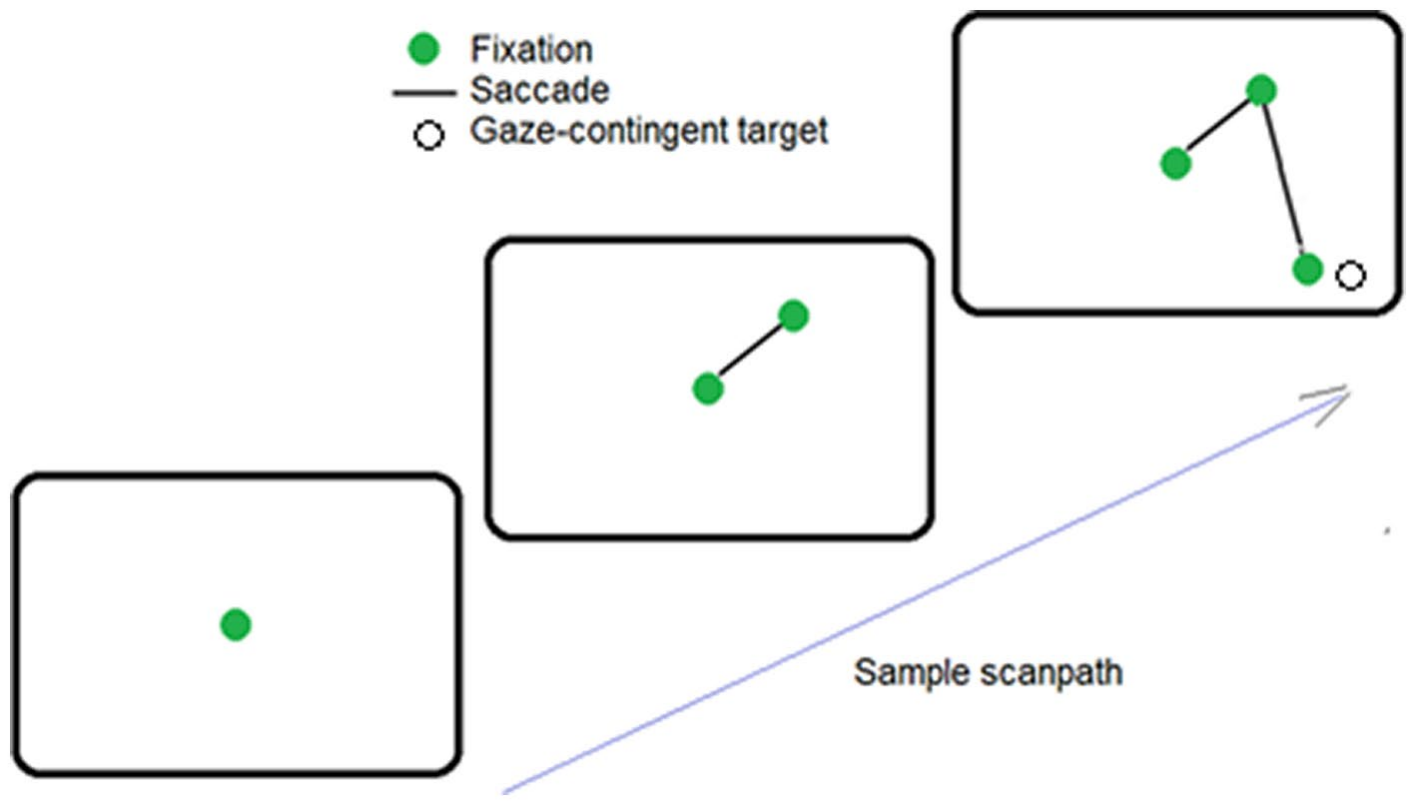
Sample sequence of saccade, when the target was found. Here, the observer fixated the center of the screen (solid green dot), and then made 2 saccades (i.e., lines). The target (an open circle) was automatically made visible when the second saccade placed fixation near its hidden position.

Participants sat 55 cm from the presentation monitor. Each session started with a 9-point calibration of the head-mounted eye tracker. This was followed by a switching off of the light (such that the only light sources were the presentation monitor and the experimenter’s eye-tracking monitor). After 2 min of dark adaptation, the experiment was started with instructions and two practice trials in which the experimenter showed the participant where to look for the difficult-to-find target. Following this familiarization phase, participants were presented with 38 search trials. An eye drift correction was performed before each trial, to maintain eye-tacking accuracy. The randomly chosen location of the target was hidden by the gaze-contingent routine, except when gaze was within 5^°^ of its location (as illustrated in Figure 1). Search trials were terminated by the participant with a mouse click if the target was found, or automatically, after 30s. The difficulty of finding the small, low-contrast target ensured that participants executed large numbers of saccades for analyses, as most trials were not terminated before the 30-s limit. An experimental session lasted 30–35 min.

## Results

### Data pre-processing

First, towards ecological validity of the search task, it was important to assess whether participants were aware of its gaze-contingent nature (i.e., the target was never visible unless gaze was directed near its location). Post-session debriefing indicated that no participant reported being aware of the gaze-contingent manipulation. The occasional reinforcement of finding the target was sufficient to motivate participants to keep searching for the hard-to-find target in ensuing trials. In effect, from the perspective of participants, the gaze-contingent display change was seamless.

Given that a goal of the study was to determine statistics of visual exploration, it was important to minimize the contributions of processes that may not reflect the acquisition of new information. Before analysis, for each participant, the initial fixation on the fixation spot, the final fixation interrupted by a trial-terminating mouse-click response, very brief fixations (<90 ms) and very small saccades (< 0.5deg) were filtered from the data. This left a mean of 1,359 (standard deviation, 651) or a median of visual exploration saccades available for analysis (median, 1,273).

### Saccades were more likely to be directed up, than down

**Figure 2:**
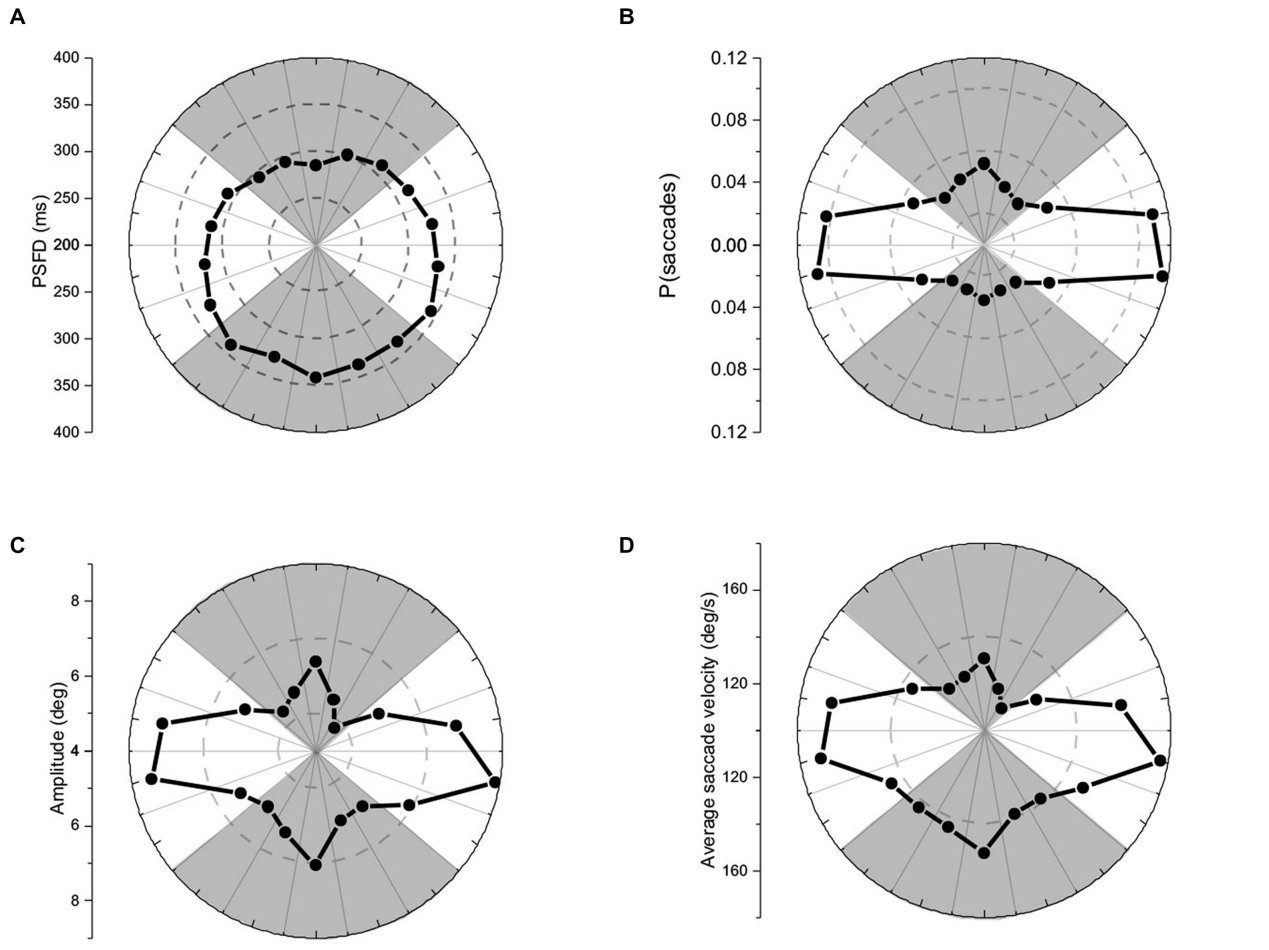
Polar plots of **(A)** pre-saccadic fixation duration, **(B)** probability of saccades, **(C)** saccade amplitude, and **(D)** average saccade velocity. The panels show respectively, earlier release, higher probability, smaller amplitude, and slower velocity for up-directed saccades during visual exploration.

To compare individual patterns of exploration irrespective of total number of saccades executed, saccade frequencies were converted to relative frequencies. Relative frequencies (i.e., probabilities) of saccades were placed in 20 deg. bins (to match the 360 deg. visual field), and analyzed using an 18- level, one-way repeated-measures ANOVA. Saccades were not equally probable in different directions, *F*(17,323) = 63.06, *p* < 0.01, ηp^2^ = 0.77 (see Figure 2B). Planned repeated-measures contrasts conducted to compare probabilities of up vs. down-directed saccades (see shaded regions of Figure 2B) indicated significantly higher probability of up- over down-directed saccades, [*t*(19) = 4.47, *p* < 0.01, two tailed, Cohen’s *d* = 0.99]. For left- vs. right-directed saccades (see non-shaded regions of Figure 2B), there was no significant difference, [*t*(19) = 1.43, *p* = 0.17, two tailed, Cohen’s *d* = 0.32].

### Saccadic asymmetries: earlier up-directed saccades

Pre-saccadic fixation durations (PSFDs) were placed in 20 deg. bins (to match the 360 deg. visual field), and analyzed using an 18- level, one-way repeated-measures ANOVA. PSFDs were not equally long in different directions, *F*(17,323) = 10.23, *p* < 0.01, ηp^2^ = 0.35 (see Figure 2A). Planned repeated-measures contrasts were conducted to compare PSFDs for up vs. down-directed saccades (see shaded regions of Figure 2A), and for left- vs. right-directed saccades (see non-shaded regions of Figure 2A). PSFDs were significantly briefer for up- than down-directed saccades, [*t*(19) = 5.89, *p* < 0.01, two tailed, Cohen’s *d* = 1.33]. PSFDs were unexpectedly longer for right- than left-directed saccades, [*t*(19) = 2.77, *p* = 0.01, two tailed, Cohen’s *d* = 0.62].

### Saccade amplitudes were smaller for up-directed saccades

Saccade amplitudes were placed in 20 deg. bins (to match the 360 deg. visual field), and analyzed using an 18- level, one-way repeated-measures ANOVA. Amplitudes were not equally large in different directions, *F*(17,323) = 15.28, *p* < 0.01, η*p*^2^ = 0.45 (see Figure 2C). Planned repeated-measures contrasts were conducted to compare amplitudes of up vs. down-directed saccades (see shaded regions of Figure 2C), and left- vs. right-directed saccades (see non-shaded regions of Figure 2C). Amplitudes were significantly smaller for up- than down-directed saccades, [t(19) = 2.69, p = 0.01, two tailed, Cohen’s d = 0.61] and not significantly different for left- and right-directed saccades, [t(19) = 0.40, *p* = 0.69, two tailed, Cohen’s d = 0.10].

### Average saccade velocities^*^ were slower for up-directed saccades

Average velocities of saccades (i.e., saccade amplitude/saccade duration) were placed in 20 deg. bins and analyzed using an 18- level, one-way repeated-measures ANOVA. Saccades were not equally fast in different directions, *F*(17,323) = 22.00, *p* < 0.01, ηp^2^ = 0.54 (see Figure 2D). Planned repeated measures contrasts conducted to compare average velocities of up vs. down-directed saccades (see shaded regions of Figure 2D) indicated significantly slower saccade velocities for up- than down-directed saccades, [*t*(19) = 5.66, *p* < 0.01, two tailed, Cohen’s *d* = 1.27]. There was no significant velocity difference between left- and right-directed saccades (see non-shaded regions of Figure 2D), [*t*(19) = 0.71, *p* = 0.49, two tailed, Cohen’s *d* = 0.17].^2^

### Kinematics: velocities of up-directed saccade were slower for amplitude-matched saccades

**Figure 3:**
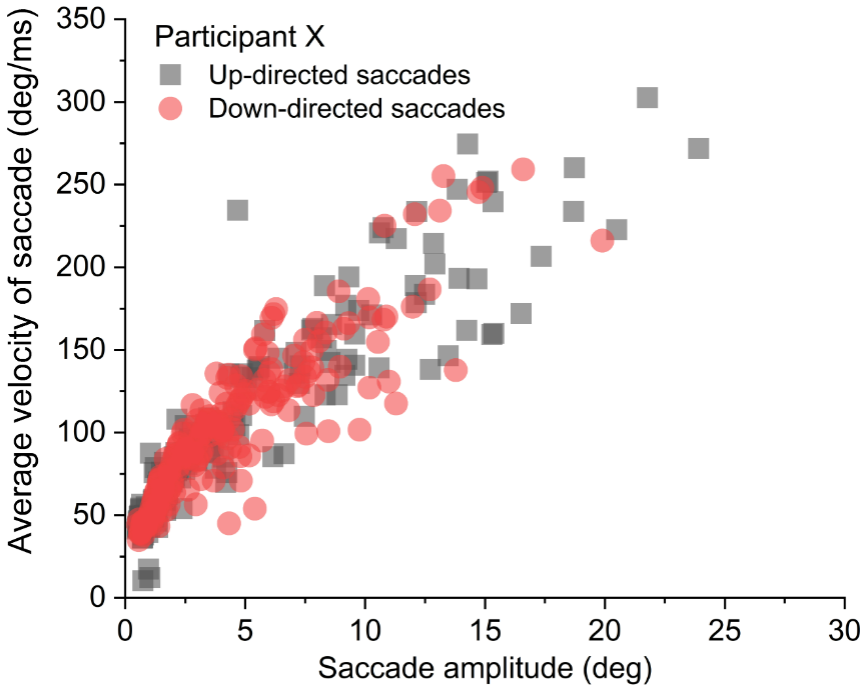
Sample data from an individual participant. For analysis, each participant’s up- and down-directed saccade data were fitted to a square root function to quantify average velocity as a function of saccade amplitude.

**Figure 4:**
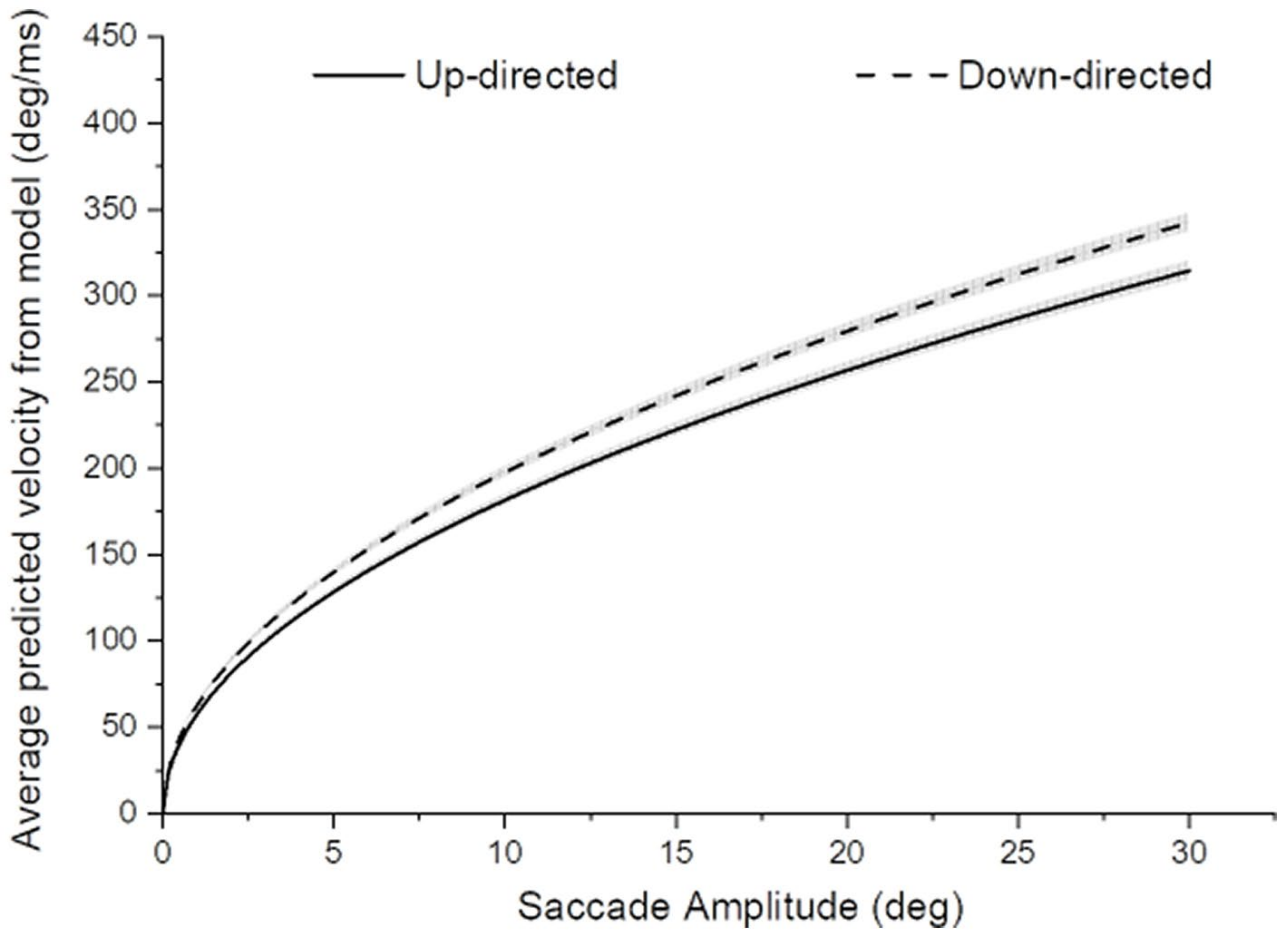
Average saccade velocity as a function of saccade amplitude for up- and down-directed saccades. The width of the curves depict ±1SE of the mean curve value. For amplitude-matched average saccade velocities, saccades tended to be slower for up- than down-directed saccades.

It was important to determine if average saccade velocities were confounded by saccade amplitude for up- vs. down-directed saccades (i.e., were up-directed saccades slower on average merely because they tended to be smaller in amplitude?). To determine kinematic relationships, each participant’s data were plotted (as shown for one participant, in Figure 3). For each participant, up-directed and down-directed saccade data were fitted to a one-parameter square root model (Velocity = S*[Saccade Amplitude]^0.5^), which has been shown to converge well with saccade kinematics (Lebedev et al., 1996; Gibaldi and Sabatini, 2021). In this model, ***S*** is a scaling parameter that shifts the function up and down along the velocity axis. Hence, higher values of ***S*** indicate faster average velocities across saccade amplitudes. The aim of the fitting procedure was to find the ***S**s* which best describe each participant’s up- and down-directed saccade data. Fitting was accomplished by the Levenberg–Marquardt algorithm in OriginLab software. Visual inspection of model fits suggested that the square root model was appropriate for the data (see also Lebedev et al., 1996; Gibaldi and Sabatini, 2021). To quantify the goodness of model fitting, the coefficient of determination statistic (***R***^**2**^) was utilized (e.g., Gibaldi and Sabatini, 2021), where good fits are reflected in values close to 1.00. Each participant’s ***S*** and ***R***^**2**^ is shown in Table 1. For quality control, a statistical comparison of ***R***^**2**^ values for up-, and down-directed saccade fits was conducted. The model fit values were comparable for both saccade directions [***R***^**2 =**^ **0**.88 vs. 0.88, *t*(19) = 0.58, *p* = 0.28, two tailed, Cohen’s *d* = 0.13]. For 16 of the 20 participants (i.e., 80%), ***S*** was higher for down-directed saccades. The mean ***S*** value was significantly higher for down-directed saccades, with a sizable Hedge’s *g* effect size [62.6 vs. 57.5, *t*(19) = −4.37, *p* < 0.01, two tailed, Cohen’s d = −0.98]. For illustration of the saccade kinematics, the curves in Figure 4 depict the average of the fitted curves. Average saccade velocity was faster for down-directed saccades.

**Table 1:** Scaling parameter value *S*, for each participant’s data fitted to a square root function.

Participant	Scaling parameter (*S*) Up-directed saccades	*R* ^2^	Scaling parameter (S) Down-directed saccades	*R* ^2^	Parameter difference
RP1	62	*0.92*	63	*0.87*	−1
RP2	59	*0.93*	67	*0.91*	−8
RP3	56	*0.90*	60	*0.92*	−4
RP4	53	*0.74*	62	*0.92*	−9
RP5	51	*0.89*	61	*0.93*	−10
RP6	49	*0.91*	59	*0.92*	−10
RP7	59	*0.78*	65	*0.78*	−6
RP8	69	*0.82*	67	*0.86*	*2*
RP9	58	*0.92*	63	*0.91*	−5
RP10	57	*0.94*	60	*0.89*	−3
RP11	59	*0.92*	66	*0.91*	−7
RP12	56	*0.94*	61	*0.93*	−5
RP13	56	*0.87*	59	*0.77*	−3
RP14	59	*0.88*	62	*0.90*	−3
RP15	56	*0.91*	66	*0.96*	−10
RP16	64	*0.88*	64	*0.79*	*0*
RP17	59	*0.87*	53	*0.81*	*6*
RP18	56	*0.90*	71	*0.84*	−15
RP19	53	*0.91*	66	*0.91*	−13
RP20	58	*0.83*	57	*0.77*	*1*
	Mean Up = 57.5		Mean Down = 62.6		

Higher values of ***S*** indicate that the data fitted a curve that was higher on the average velocity axis. The ***R***^**2**^ (coefficient of determination) values represent goodness of fit of the data to the fitting function (higher values indicate very good fits).

## Discussion

The first goal of the study was to determine vertical asymmetries in PSFDs, saccade amplitude, saccade probability, and average saccade velocity during visual exploration, devoid of confounding clutter in the visual field. Saccade kinematics (specifically, saccade velocities as a function of saccade amplitude) were also determined. To accomplish the first aim, gaze-contingent eye-tracking technology was utilized to keep the search display clutter-free, and the target was made visible only when a saccade landed near its hidden location. The second goal was to introduce a theory of vertical saccade inhibition, towards an explanation of all the saccadic results.

Importantly with respect to the first aim, the gaze-contingent manipulation did not introduce any unwanted distraction, as no participant reported being aware of gaze-contingency in the search displays. Replicating previously-reported vertical asymmetries, up-directed saccades were executed earlier (Foulsham and Kingstone, 2010; Greene et al., 2014; see also Greene et al., 2020, were faster (Bonnet et al., 2013; Tiadi et al., 2014), were smaller in amplitude (Greene et al., 2010), and more probable (Foulsham and Kingstone, 2010; Greene et al., 2014) than down-directed saccades. The finding of earlier left- than right-directed saccades was unusual (c.f. Greene et al., 2014 where no horizontal asymmetry was apparent in 3 experiments). It is possible that the lack of uncontrolled clutter implemented in the present task contributed to this finding. It may also possibly be explained by a statistical Type I error. Future experiments must be conducted with a similar paradigm to test the robustness of this finding. No significant horizontal asymmetry was found, otherwise. Finally, for amplitude-matched average saccade velocities, saccades tended to be slower for up- than down-directed saccades. Based on vertical asymmetry results and insights from neural control of saccades, an ecologically motivated theory of vertical saccade inhibition is presented to fulfill the second goal of the study.

### Neural control of saccades: an overview

**Figure 5:**
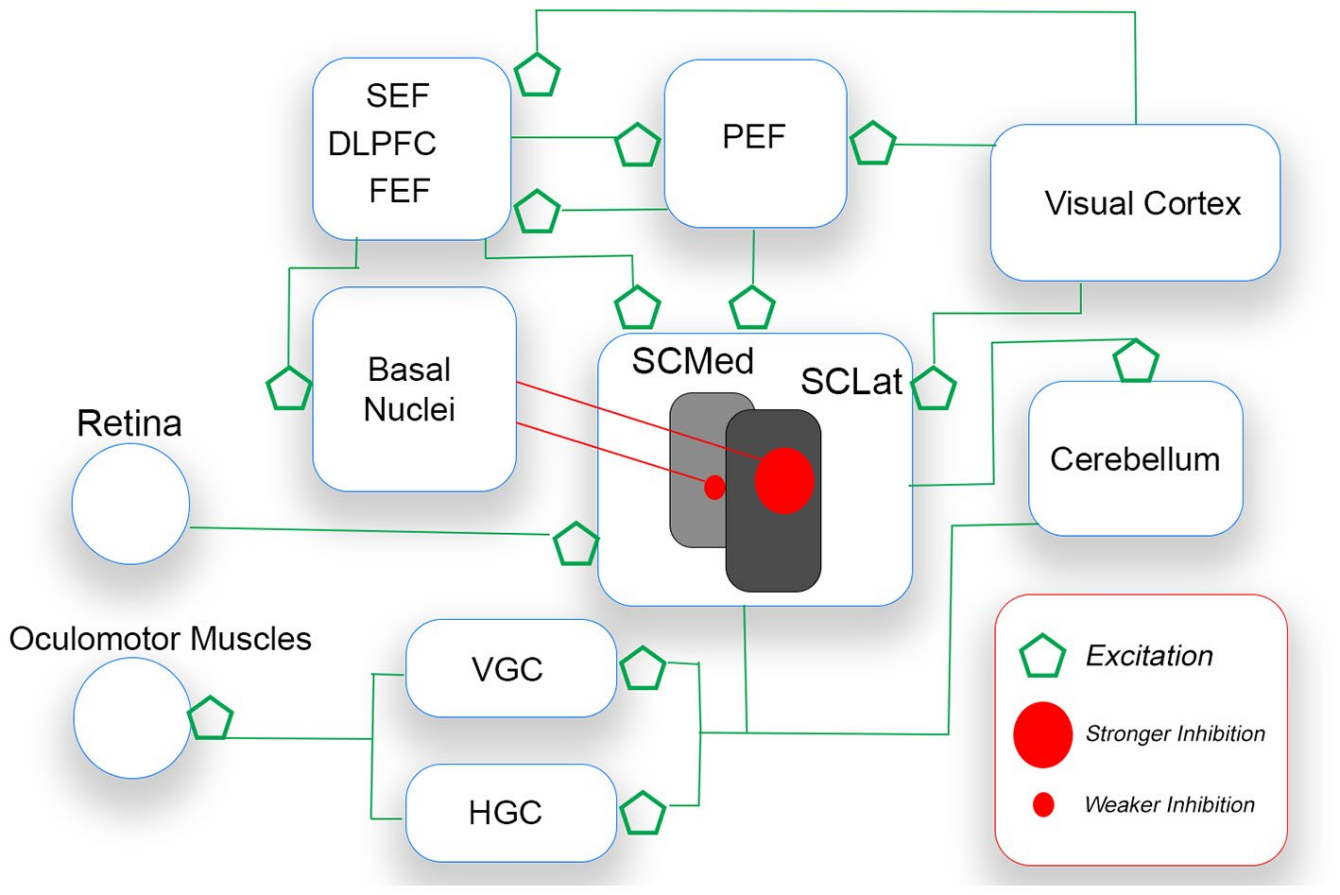
A simplified schematic of the voluntary saccade circuitry. Saccades are initiated when the gaze centers (VGC and HGC) stimulate oculomotor muscles of the eyes. The SC which sends saccade commands to VGC and HGC receives excitatory signals from bottom up (e.g., retina, visual cortex) and top-down (e.g., FEF, PEF) sources. As only one saccade is possible at a time, the basal nuclei (BN) inhibit the releasing of SC command signals. The BN release the SC from inhibition, after an appropriate saccade target is selected. The theory proposed is that pre-saccadic inhibition is stronger for down-directed saccades (controlled by lateral regions of the SC) than for up-directed saccades.

For humans, the external world is characterized by competing demands in the visual field. Saccades are necessarily executed along horizontal and vertical axes. A planned saccade vector is controlled by an unfolding and complex interplay among cerebellum, brainstem, midbrain, and cerebral cortex (Girard and Berthoz, 2005; Terao et al., 2017). Central to the execution of saccades is the superior colliculus (SC), a primitive structure in the midbrain that receives input from the retina, basal nuclei, and many cerebral regions (i.e., visual cortex, dorsolateral prefrontal area, parietal, supplementary and frontal eye fields). With respect to retinal input, the SC serves as a head/eye movement reflex center. The structure also receives excitatory input from cortical sources which process competing stimulation of interest in the visual field (see Figure 5). Without restraint, the SC (i.e., this head/eye movement reflex center) would continually trigger a saccade to salient visual stimulation, irrespective of the stimulation’s relevance/importance to the observer. To ensure saccadic control, the SC is placed under sustained inhibition by the basal nuclei (BN), permitting individual saccades to be executed in the face of competing saccadic demands (Hikosaka et al., 2000; Terao et al., 2017; Macpherson and Hikida, 2019). Thus, a saccade is released (mostly to capture meaningful targets) when there is a reduction in SC inhibition from the BN (Hikosaka et al., 2000).

Different regions of the SC are responsible for different kinds of saccadic activity. Rostrally-located regions control smaller saccades (e.g., fixation saccades, microsaccades), and more caudally-located regions control larger saccades. Most important for the present discussion is that, on balance, medial SC regions dominate the releasing of *up*-directed saccades *from sustained inhibition*, while lateral SC regions dominate the releasing of *down*-directed saccades (e.g., Robinson, 1972; Hafed and Chen, 2016). The released SC stimulates gaze-center neurons in the brainstem. The medial portion of the paramedian pontine reticular formation (PPRF, or horizontal gaze center,) is dominant for the horizontal component of the saccade vector, and the rostral interstitial nucleus of the midbrain reticular formation (rinMRF, or vertical gaze center) is dominant for the vertical component (e.g., Sparks, 2002). Gaze-center neurons stimulate motor neurons connected to oculomotor muscles that move the eyes.

### Ecological theory of asymmetric vertical saccade inhibition

Saccades dominate gaze shifts within the foveal, parafoveal and near peripheral visual field of eye fixation (Freedman, 2008; Franchak et al., 2021). Assuming that the saccadic system is efficient, it is logical to expect it to process differently, signals that are more frequent in occurrence (see Simoncelli and Olshausen, 2001 for a review of natural image statistics and neural representation). The A-VSI theory is based on the assumption that the saccadic system is adaptive and learns to, or has evolved to respond efficiently to regularities of input from the upper and lower visual fields (see Previc, 1990 for a proposal of ecological origins of functional specialization in the upper and lower visual fields of humans).

With an upright torso, peri-personal space is typically *below* eye fixation (i.e., in our lower visual field). Hence, targets in peri-personal space typically require a *down*-directed saccade. Extra-personal space, in contrast, is beyond arm’s reach, and is typically *above* eye fixation (i.e., in our upper visual field). Luminance contrast sensitivity (Skrandies, 1987; Levine and McAnany, 2005; Abrams et al., 2012; Silva et al., 2018), and temporal contrast sensitivity (Skrandies, 1987; Kremláček et al., 2004; Levine and McAnany, 2005) are higher in the lower visual field. In effect, within matched visual angles around eye fixation, afferent stimulation from the lower visual field is more intense than stimulation from the upper visual field. In a busy visual field (with sustained and transient stimulations), this creates a bias for reflexive saccade triggers towards the lower visual field. Given the asymmetric positioning of the human head atop the upright torso, the lower visual field afferents are susceptible to greater distraction (e.g., from limbs, near-torso objects, ground level optic flow) for reflexive saccade triggers than the upper visual field afferents. Indeed, optic flow in the lower visual field is more disruptive (i.e., induces stronger vection) than optic flow in the upper visual field (e.g., Fujimoto and Ashida, 2019). Whereas specialization of the upper visual field processing promotes earlier releasing (i.e., shorter latencies; see Zhou and King, 2002, Tzelepi et al., 2010, Greene et al., 2014, Abegg et al., 2015), slower moving (e.g., Bonnet et al., 2013; Tiadi et al., 2014), smaller in amplitude, and more frequent saccades, a formal mechanism for the asymmetries has not been proposed. In the present article, given the centrality of the SC to the execution of saccades, it is proposed that asymmetrical sustained inhibition of the SC is responsible for the asymmetries observed.

### Contradictions between saccadic versus manual reactions?

Curiously, the asymmetry typically reported for manual reaction time (MRT) tasks (Maehara et al., 2004; Kokubu et al., 2018) is opposite to that reported for saccade latencies. In MRT tasks, latencies are *longer* when the cue appears in the upper visual field. By comparison, MRTs are typically longer than saccade latencies in reaction time tasks, and may be governed by different functions (Jaśkowski and Sobieralska, 2004; Gambacorta et al., 2018). Within the ecological theory proposed in the present work, MRT and saccade latency patterns may be reconciled by the findings of Kokubu et al. (2018). In their study, MRTs of participants were recorded under four fixation distances on a horizontal table. What is notable for the present discussion, is that Response Cue Location (UpVF vs. LoVF) interacted with Fixation Distance (Near, Middle, Far, Very far) such that the MRT asymmetry was increasingly reduced with greater fixation distances. In effect, the farther away participants fixated from their torso, the more the MRT asymmetry approached the direction of the saccade latency asymmetry. Kokubu et al.’s (2018) data suggest that the typically-reported faster MRTs for LoVF stimuli is not independent of ecological constraints.

### Future perspectives

In this paper, it has been argued that because of the great potential for distraction in the lower visual field, it is important to resist looking down. In line with an efficient coding theory (e.g., Simoncelli and Olshausen, 2001), it is reasonable to expect lateral SC regions which dominate the releasing of *down*-directed saccades to be under stronger sustained inhibition than medial SC regions. According to A-VSI, it is easier to overcome sustained inhibition of upper visual field saccade programs. Hence, for up-directed saccades, neural processing is stronger, occurs earlier (Hafed and Chen, 2016), is released more frequently (Greene et al., 2014 the present study), and is released earlier (Zhou and King, 2002; Abegg et al., 2015; Greene et al., 2020; the present study). A speculation on the slower average velocity for up-directed saccades also comes from A-VSI. If up-directed saccade programs are less inhibited, there may be less of a buildup of antagonistic activity to overcome, resulting in slower velocities. Smaller up-directed saccade amplitudes may also be indirectly linked to asymmetric inhibition of the SC. During visual exploration, useful information for locating and identifying objects is available within a perceptual span around the fixation point (Rayner, 1998; Rayner, 2009). There is greater nearby distraction in the lower visual field (given the positioning of the head atop the torso) and this distraction is processed with greater contrast sensitivity (Abrams et al., 2012; Silva et al., 2018). On average, saccades tend to move fixations just outside the perceptual span (Motter and Belky, 1998). Given the larger perceptual span in the lower visual field (e.g., Greene et al., 2010), reasonably, saccades into the lower visual field would be more likely released towards more distant (i.e., less processed) distractors.

The vertically asymmetric inhibition described in this article may be tested more directly with anti-saccade tasks (see Coe and Munoz, 2017 for a discussion of saccade inhibition in anti-saccade tasks). In an anti-saccade task, the observer must fixate a stimulus and then make a saccade to the mirror location of an attracting peripheral target. Anti-saccade latencies are longer compared to prosaccade latencies (i.e., making a saccade towards the attracting peripheral target). As saccade-optimized neuronal activity in the SC is direction-sensitive, the balance of activity is biased towards the visible target, thus hindering saccade initiation to the mirror (anti-saccade) location of the target (Munoz and Fecteau, 2002). The hinderance is manifested as saccade direction errors and delayed saccade reaction time when a directional error is absent. An open question is whether there is an asymmetry in hindrance for vertical anti-saccades. According to the A-VSI theory, there should be strong inhibition for the programming of down-directed prosaccades (cued by an attracting peripheral target below eye fixation), and weak inhibition for the programming of up-directed prosaccades (cued by an attracting peripheral target above eye fixation). Hence, it should be more difficult to inhibit an *anti-saccade* cue above than below fixation. The A-VSI theory predicts a greater hindrance for down-directed anti-saccades (cued by a weakly inhibited distraction above eye fixation) than for up-directed anti-saccades (cued by strongly inhibited distraction below eye fixation). This prediction will be tested in future studies.

The term “eye fixation” is a misnomer, as the eyes are never perfectly still (i.e., fixated). Another test of the A-VSI theory may come from directional behavior of microsaccades that occur during a prolonged (i.e., 60s) eye fixation (e.g., Hermens and Walker, 2010). Fixation microsaccades occur while saccades in all directions are placed under sustained inhibition. The A-VSI theory reasonably predicts greater inhibition of down-directed than up-directed microsaccades. Published polar plots suggest that during prolonged eye fixations, human binocular up-directed microsaccades may be more prevalent (i.e., more easily released) than down-directed microsaccades (see Figure 4 in Denison et al., 2019 and Figure 4 in Hermens and Walker, 2010). The work of Denison et al. (2019) is particularly interesting for future research. They showed informally, that microsaccades tend to be more frequently released upwards than downwards when they occur just before the predictable onset of a transient target. These pre-target microsaccades also exhibit greater fixation stability than earlier microsaccades, and are indicators of inhibitory influences on the execution of microsaccades (see Denison et al., 2019). To date, directional metrics of microsaccades have not been determined in a formal manner (i.e., with statistical inferences). The A-VSI theory may motivate future studies on microsaccades.

Many other open questions remain. An important limitation of the present work is that while the theory is logical in its proposal of inhibition, the physiological mechanism of inhibition (by BN and cortical structures) is not addressed. Interestingly, the ecologically-motivated asymmetric SC inhibition principle works beyond vertical saccades. For example, Koller and Rafal (2019) hypothesized from previous data, that express saccades would be released earlier for temporal field over nasal field triggers. The asymmetric inhibition mechanism proposed in the present article would allow for the same hypothesis. Given the asymmetric positioning of the nose with respect to any one eye (and hence a regular peri-personal distractor in the nasal field), it would be efficient to more strongly inhibit distracting saccades triggered from the nasal side of the field. Indeed, Koller and Rafal (2019) found earlier saccades directed towards the temporal than nasal visual field, in support of greater inhibition of saccade programming in the nasal field.

The proposed theory is testable within many domains of the NIH’s Research Domain Criteria (RDoC) initiative designed for the investigation of mental processing and disorders (see Harrison et al., 2019). RDoC consists of many constructs that define an overall range of functions (i.e., behavioral elements, processes, mechanisms, and responses). To date, the constructs are categorized within 6 domains (i.e., Negative valence, Positive valence, Cognitive, Social processes, Arousal/Regulatory, and Sensorimotor). The investigation of domain issues is encouraged among multiple units of analysis (genes, molecules, cells, neuro-circuits, physiology, behaviors, and self-reports). In the present context, research may target the Attention construct of the Cognitive domain, and the Motor action construct of the Sensorimotor domain. Investigations may utilize paradigms that allow for the observation of vertical saccadic activity. Relevant RDoC units of analysis include molecules (e.g., Glutamate and GABA levels), neuro-circuits (e.g., functional and neurochemical imaging), physiology (e.g., ERP, MEG), and behaviors (e.g., saccade reaction times, presaccadic fixation duration during visual search, saccade velocity, microsaccade metrics during prolonged fixation).

## Conclusion

To conclude, the results of the present study support previous findings of earlier, slower, smaller, and higher probability of up-directed saccades during visual exploration. An ecological theory of asymmetric inhibition of saccadic programs was introduced to explain the results. The present study demonstrates the usefulness of exploiting asymmetries in vertical saccadic metrics in healthy volunteers. This kind of study has substantial implications for clinical disorders like schizophrenia and bipolar disorder wherein alterations in the saccadic system are associated with clinical symptoms such as psychosis and mania (Bittencourt et al., 2013). The unique characteristics of vertical saccade metrics may contribute to increased diagnostic specificity and provide new insights into the limits of saccadic impairments (Thakkar et al., 2017).

## Data availability statement

The raw data supporting the conclusions of this article will be made available by the authors, without undue reservation.

## Ethics statement

The studies involving human participants were reviewed and approved by University of Detroit Mercy IRB (protocol IRB-C#18–19-30). The patients/participants provided their written informed consent to participate in this study.

## Author contributions

HG: conceptualization, methodology, software, data collection, and writing—original draft preparation. VD: writing—original draft preparation and writing—reviewing and editing. JB: conceptualization and writing—reviewing and editing. All authors contributed to the article and approved the submitted version.

## Conflict of interest

The authors declare that the research was conducted in the absence of any commercial or financial relationships that could be construed as a potential conflict of interest.

## Publisher’s note

All claims expressed in this article are solely those of the authors and do not necessarily represent those of their affiliated organizations, or those of the publisher, the editors and the reviewers. Any product that may be evaluated in this article, or claim that may be made by its manufacturer, is not guaranteed or endorsed by the publisher.

## Footnotes

^1^ http://blogs.umass.edu/eyelab/software/

^2^*While peak velocity is more commonly reported than average velocity, a very strong linear relationship exists between the two variables (*r* > 0.90; see Lebedev et al., 1996).
